# Exploration and Analysis on the Psychological Capital of Entrepreneurship and the Deviant Innovation Behavior of Employees

**DOI:** 10.3389/fpsyg.2020.01880

**Published:** 2020-08-04

**Authors:** Qianying Gao, Jing Xu, Zhe Tao, Li Liu, Cisheng Wu

**Affiliations:** ^1^School of Management, Hefei University of Technology, Hefei, China; ^2^School of Humanity and Law, Hefei University of Technology, Hefei, China

**Keywords:** psychological capital, entrepreneurial opportunity ability, deviant innovation behavior, entrepreneurial performance, emotional intelligence

## Abstract

This study aims to explore the psychological capital of entrepreneurship and the deviant innovation behavior of employees, thereby providing theoretical support for the implementation of Chinese innovation policy. By analyzing the previous research results, this study used questionnaires to collect the required data for exploration. Two questionnaires were designed: one was a survey for the psychological capital of entrepreneurship, and the other was a survey for deviant innovation behavior of employees. The research results show that the correlation coefficient between entrepreneurial performance and entrepreneurial psychological capital is 0.29, indicating that there is a significant correlation between entrepreneurial psychological capital and entrepreneurial performance; the correlation coefficient between entrepreneurial opportunity ability and entrepreneurial performance is 0.40, indicating that there is a significant correlation between entrepreneurial opportunity ability and entrepreneurial performance; the correlation coefficient between entrepreneurial psychological capital and entrepreneurial opportunity ability is 0.81, indicating that there is a significant correlation between entrepreneurial psychological capital and entrepreneurial opportunity ability. In addition, there are significant correlations between the work values and psychological empowerment of employees, the work values and deviance innovation of employees, and the psychological empowerment and deviance innovation of employees. The scores of psychological empowerment and work values of employees during the period of low emotional intelligence show a downward trend, while the scores of the psychological empowerment of employees increase significantly when they show high emotional intelligence, and the differences are statistically significant. When task interdependence is high, the psychological empowerment and deviance innovation scores of employees are lower, and the difference is statistically significant. For this purpose, this study enriches the research on psychological capital and deviant innovation and provides a reference for the practice of innovation management.

## Introduction

With the support and encouragement of Chinese “Mass Entrepreneurship and Innovation” policy, the number of innovative entrepreneurs has increased significantly. Entrepreneurship is one of the important factors to promote economic development, and innovation is also the source of economic development in China. Both are indispensable in the process of economic and social development in China (Huang et al., 2018; Wang et al., 2018). However, there are very few successful entrepreneurs, which makes people wonder why some entrepreneurs may succeed. Why do some people succeed? What do successful entrepreneurs have in common? Such questions may have varied answers, but the common point is that there is no exception to the identification of entrepreneurial psychological capital ability that affects entrepreneurial success (Zollo et al., 2018). In addition to entrepreneurs, innovators have now become the object of strong national support, but compared to the process and the methods of innovation, the government or the enterprise attaches more importance to the results of innovation (Neumeyer et al., 2019). At present, there is a situation where a company expects its employees to innovate to some extent and encourages them to do so. However, if the creative thinking of employee conflicts with the development goals of the company, the innovative ideas of employees will be rejected with a high probability. This will greatly crack down on the innovative ideas of employees; yet if employees insist on their innovative ideas and continue to innovate and unfold the ideas secretly, they will be considered as performing deviant innovation behaviors. Therefore, this study explores the psychological capital of entrepreneurship and deviant innovation behavior of employees separately.

At present, the Chinese research in the theoretical circles on work remodeling and innovations has just started. Even so, it is not difficult to find that the research on work reshaping is not yet in-depth, and some key issues have not been effectively resolved. On this basis, for the purpose of exploring the entrepreneurial psychological capital and employee deviant innovation behaviors, as well as analyzing the relation between entrepreneurial psychological capital and employee deviant innovation behaviors, the enterprise employees were taken as the research objects. It is expected to enrich the research on entrepreneurial psychological capital and deviant innovation behaviors, thereby providing a reference for the innovation management practices of enterprises.

## Literature Review

Scholars worldwide have also made very detailed studies on these aspects. A sample survey of 369 women entrepreneurs in small and medium-sized enterprises in Gujarat in Western India by Digan et al. (2019) found that the enhancement of women’s entrepreneurial rights and business income were in positive correlation, which could strengthen the benefits of empowerment further by managing resource constraints (through capital restructuring) and psychological capital to address the challenges of self-employment. This study helped government agencies and non-governmental organizations to formulate programs and policies to improve the performance of enterprises owned by women in developing countries (Digan et al., 2019). Marashian and Naderi (2014) explored the relationship between the organizational culture, emotional intelligence, psychological capital, work self-efficacy, and organizational entrepreneurship of employees in Khuzestan Hydropower Company in Iran. Through the analysis of typical related data, it was found that employee organizational culture had significant correlations with emotional intelligence, psychological capital, work self-efficacy, and organizational entrepreneurship. Fuentelsaz and Montero (2015) and Shen and Ho (2020) researched the entrepreneurs with a high tendency to innovation through a sample survey of 65,000 entrepreneurs from 88 countries. These entrepreneurs participated in the Growth Enterprise Market project from 2008 to 2012. The research results showed that from a psychological perspective, the most important variables included risk tolerance, entrepreneurial alertness, and confidence in skills, and education and past experience also played a key role in human capital. Khurosani (2018) and Zheng et al. (2020) used structural equation modeling to conduct organizational learning culture research on managers and employees of the creative industry in Banten, Indonesia. The research results showed that transformation leaders had a positive impact on organizational learning culture but did not affect the creativity and organizational innovation of employees; organizational learning culture had a positive impact on employee creativity; employee creativity had a positive impact on organizational innovation.

Through analysis on the existing research, it is found that the current research on deviant innovation still has the following shortcomings. First, the existing literature studies and measures on the two manifestations of deviant innovation behavior are completely separate, which is regarded as two separate processes. Based on whether managers are aware of this perspective, “private innovation” is precisely the off-track innovation behavior that occurs before managers are aware of it, while “illegal innovation” is an off-track innovation behavior that occurs after managers are informed. The innovation process will change from a private state to an open state. Thus, two deviant innovations can form a dynamic innovation process. Therefore, the existing measurement methods for employees’ deviant innovation behaviors are not conducive to an accurate understanding of deviant innovation behaviors. Second, the existing research on deviant innovation behaviors only sporadically proposes the influencing factors of deviant innovation behaviors at the individual, leadership, and organizational levels, lacking the disclosure of action mechanisms and the support of empirical results. Especially, the research on the formation mechanism of deviant innovation behavior in Chinese context is rare. This is not conducive to industrialists and scholars to have a deep understanding of the formation process of deviant innovation behavior. Third, most of the existing research focuses on the value that the success of deviant innovation will bring to the organization, as well as the negative impact that the failure of deviant innovation will bring to the organization. However, as an unconventional employee behavior, its impact on the organization is often more complicated than conventional behavior (Yu et al., 2019). The dual attributes of lawful purpose and behavior deviation also lead to successful and failed deviant innovation behaviors, which will have opposite effects. Therefore, it is necessary to explore the negative (positive) influence of successful (failed) deviant innovation behavior. In addition, most previous studies have focused on the impact of deviant innovation on individual innovation performance, ignoring its impact on organizational-level variables, especially its impact on the ultimate goal of organizational development-organizational effectiveness.

## Materials and Methods

### Psychological Capital of Entrepreneurship

Psychological Capital Appreciation (PCA) refers to a positive psychological state manifested by individuals during their growth and development. It is a core psychological element that transcends human and social capitals as well as a psychological resource that promotes personal growth and performance improvement. With the increasing pressure of competition and the speed of changes in modern enterprises, more enterprise managers realize that employees generally have work pressure and ideological burdens. They pay attention to salary and benefits but pay more attention to growth and progress. They can accept the pressure of heavy work and lower payment, but they are in urgent need of psychological comfort (Lerner et al., 2019).

The entrepreneurial psychological capital is an extension of the concept of PCA, targeting the psychological resources and psychological qualities of the entrepreneurial crowd. According to the 7 elements of entrepreneurial psychological capital - active growth, proactive response, enthusiasm and innovation, keen excellence, self-efficacy, social wisdom, and optimistic hope, the entrepreneurial psychological capital of entrepreneurs is studied. Active growth mainly describes the views of entrepreneurs on life, selection of goals, and the ability to execute self-awareness. Active response mainly describes the ability of entrepreneurs to cope with difficulties and frustrations, the practices when encountering difficulties, and the continuous improvement and update of their knowledge structure. Enthusiastic innovation mainly describes the attitude of entrepreneurs to life and entrepreneurship. Entrepreneurs should maintain their vitality in the process of entrepreneurship to accept various challenges from the workplace. Sensitive excellence mainly describes whether entrepreneurs can achieve critical thinking when faced with the market and are keen to identify market needs. Self-efficacy mainly describes the ability of entrepreneurs to deal with problems and whether they can face the challenges by themselves. Social wisdom mainly describes whether entrepreneurs can empathize, understand their needs while being aware of the thoughts and needs of others, and make correct judgments and actions. At the same time, they must have strong communication skills to win various opportunities. Optimistic hope mainly describes whether the mentality of entrepreneurs can be maintained peacefully and whether they can remain rational and hope in the face of setbacks. In summary, it is not difficult to find that entrepreneurial psychological capital has a great impact on entrepreneurs. Therefore, in the study of innovative entrepreneurial behavior, it is necessary to discuss entrepreneurial psychological capital.

### Entrepreneurial Opportunity Ability

Entrepreneurial opportunity ability mainly refers to the attractive and long-lasting business opportunities that are conducive to entrepreneurship. Entrepreneurs can provide customers with valuable products or services and benefit themselves at the same time. An entrepreneurial opportunity is a product or service that creates or adds value to a buyer or user (Rogoza et al., 2018), and it is attractive, durable, and timely. An entrepreneurial opportunity introduces a new product, new service, new raw material, and new organization selling at a price higher than the cost. Entrepreneurial opportunity is a new “Means-End” relationship, which can introduce new products, new services, new raw materials, new markets, or new ways of organizing economic activities.

At present, there are two factions. One believes that entrepreneurial opportunities exist independently of entrepreneurs, and entrepreneurs need to find out the entrepreneurial opportunities from the external environment. The other holds that entrepreneurial opportunities are closely related to entrepreneurs themselves; the acquisition of entrepreneurial opportunities is based on the perception and understanding of entrepreneurs (Ramadi et al., 2019). In this study, the entrepreneurial opportunity ability is defined as the search and perception of entrepreneurs on various information in the market, identifying the potential entrepreneurial opportunity in the market, and selecting the appropriate entrepreneurial method to start their business. The entrepreneurial opportunity ability in this study mainly includes three capabilities: opportunity search, opportunity identification, and opportunity evaluation.

Opportunity search mainly reflects the sensitivity of entrepreneurs to external information, whether they can search for information that is beneficial to themselves and collect information; opportunity identification mainly reflects the ability of entrepreneurs to judge whether the collected information has market value; opportunity evaluation mainly reflects the evaluation of the relationship between an opportunity and themselves when the entrepreneurs face an opportunity and evaluate whether the opportunity is beneficial to their business. Many entrepreneurs who achieve successful entrepreneurship have been able to continue to grow and develop precisely because they have seized the opportunity. Therefore, in innovative entrepreneurship, entrepreneurship opportunities are also one of the key influencing factors that should be valued.

### Entrepreneurial Performance

Entrepreneurial performance refers to the degree to which a task or a certain goal is achieved in the process of entrepreneurship. The goal theory holds that each organization has formulated its end goal, and performance should be measured by the degree to which the goal is achieved. In entrepreneurship research, goal theory is also welcomed by scholars. Since ownership and management rights are not separated in entrepreneurship, entrepreneurs or entrepreneurial teams dominate the entrepreneurial performance. Therefore, the goal theory serves as the theoretical basis for entrepreneurial performance. There is certain rationality that is worthy of reference for people in future entrepreneurial research. However, this theory also has certain shortcomings. The measurement of targets is not as objective as what is in actual operation (Nikolaev et al., 2018). In general, there is often more than one goal for an organization. Each organization’s department has its goals. Sometimes, the goals of these departments conflict with each other. Besides, the organizations have their respective goals. How to measure these factors makes it difficult to compare the performance of various organizations, and it is impossible to distinguish the advantages and disadvantages among them (Barbasanchez and Atienzasahuquillo, 2018).

In this study, entrepreneurial performance was mainly measured from the dimensions of business operation, viability, survival time, profit duration, investment return, total sales, employee growth, and timeshare growth. Entrepreneurship performance is a direct reflection of the level and ability of entrepreneurial innovation, which is also an influential factor that cannot be ignored. Therefore, the entrepreneurial performance was introduced as an element to measure and evaluate entrepreneurial psychological capital and deviant innovation behaviors.

### Deviant Innovation

After analyzing and investigating related researches, this study summarized the characteristics of employee deviant innovation into the following points, which were also important factors that influence the innovative behaviors of employees.

(1) Privacy: Since the company disagrees with, supports, or disapproves the deviant innovation behavior of employees and their innovative ideas, while the deviant innovation behavior is not within the scope of daily work requirements of employees, the deviant innovation behavior of employees will occupy the daily working time of employees, consume their work energy, and occupy the public resources of companies. Accordingly, when the company finds deviant innovation behavior, the employees are punished. Meanwhile, employees need to ensure that their behaviors are private to avoid such punishments, which means that such deviant innovation behaviors of employees usually occur outside the line of sight of the management (Shen et al., 2019).
(2) Duality: During the deviant innovation behavior, the behavior of employees will violate the rules, regulations, and related codes of conduct of the companies. Such behaviors of the employees are illegal, even if the starting point of the deviant innovation behavior is in the interest of the companies. However, if the deviant innovation behavior of employees is unsuccessful, it will become an abnormal behavior that affects the interests of the organization and the company. Also, the starting point of deviant innovation behavior of employees is in the interests of the companies, and the purpose is to create benefits for the company, so its purpose is legitimate. Thus, the deviant innovation behavior of employees is dual.
(3) Risks: Innovation are certainly risky. Employees are also at risk of whether their innovation results are accepted, and employees’ innovation results will be directly related to their personal interests. If the company recognizes the innovation results of employees, as a result, they will get some additional benefits. If the company does not agree with the results of the innovation of employees, they will also be subject to certain departures, affecting their personal interests. It is also because of the attitude of the companies toward the results of deviant innovation behavior of employees that has led to the continuous occurrence of deviant innovations of employees. The company does not punish employees because their innovation results are the result of deviant innovation behavior. On the contrary, if their innovation results are conducive to the development of the company, the company will reward them; thus, employees will have gambler-like psychology for deviant innovations regardless of company regulations and institutional innovations (Hoogendoorn et al., 2019).

### Research Model

**FIGURE 1:**
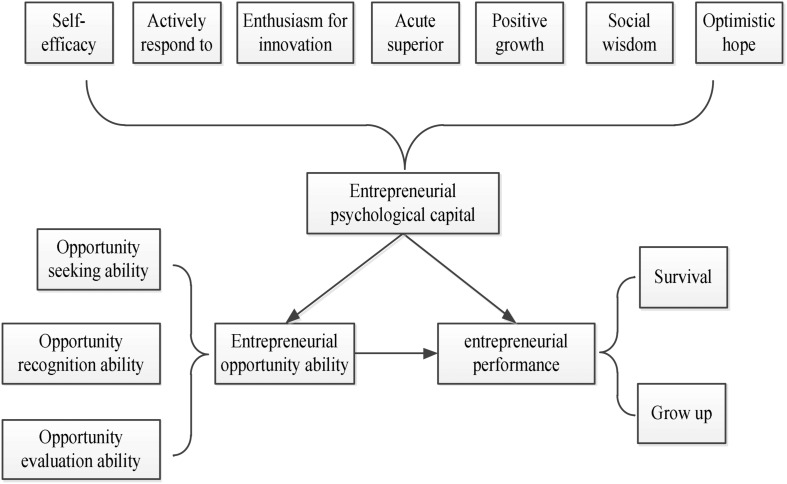
Hypothetical model of psychological capital of entrepreneurship.

**FIGURE 2:**
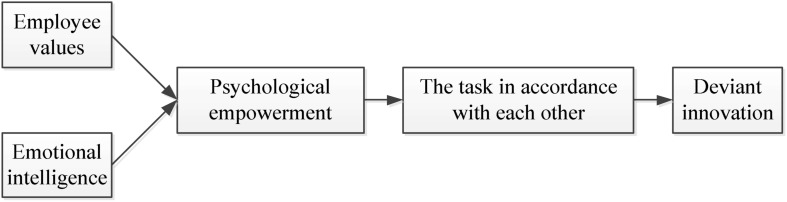
Hypothetical model of deviant innovation behavior of employees.

According to the previous research results, this study established a hypothetical model based on the psychological capital of entrepreneurship and deviant innovation behavior of employees, as shown in Figures 1, 2.

According to Figure 1, the study described the relationship between innovative psychological capital, innovation performance, and innovation opportunity capability. This study made 4 hypotheses: (1) There is positive correlation between psychological capital of entrepreneurship and entrepreneurial performance; (2) There is positive correlation between psychological capital of entrepreneurship and entrepreneurial opportunity ability; (3) There is positive correlation between entrepreneurial performance and entrepreneurial opportunity ability; (4) The entrepreneurial opportunity ability plays an intermediary role in both psychological capital of entrepreneurship and entrepreneurial performance.

According to Figure 2, this study combined the deviant innovation behavior of employees with work values, emotional intelligence, psychological empowerment, and task interdependence with the employee. Work values of employees refer to the attitudes of employees toward work and the degree of investment in work. The deviant and innovative behavior of employees is a reflection of both their work values and their work preferences, which can also demonstrate the interests of employees in pursuing the company benefits. Emotional intelligence refers to the level of employees’ understanding and cognition of other people and their emotions. Emotional intelligence helps employees adapt to various interpersonal communication environments and can better regulate their emotions. Psychological empowerment refers to the employees’ perception of how they match the job content. The psychological empowerment of employees includes their understanding of the meaning of the work, which will affect the autonomy and self-efficacy of employees. Task interdependence refers to the degree of correlation between work productivity among employees, that is, the degree of correlation between the work of employees and the work of other employees (Bajracharya et al., 2018). This study makes the following hypotheses: (1) There is positive correlation between deviant innovation behavior of employees and their work values; (2) Psychological authorization plays an intermediary role in the work values and deviant innovation behavior of employees; (3) There is a certain relationship between emotional intelligence and work values and psychological empowerment of the employees; (4) There is a certain correlation among task interdependence, psychological empowerment, and deviant innovation behavior of employees.

### Research Samples and Data

A questionnaire survey was conducted to collect the required data for exploration. In this study, two questionnaires were designed: one was a survey for the entrepreneurial psychological capital of entrepreneurs (Questionnaire 1), and the other was a survey for deviant innovation behavior of employees (Questionnaire 2). Questionnaires were distributed to 4 cities including Shanghai and Nanjing through the Internet. For Questionnaire 1, a total of 350 copies were sent out and 317 were retrieved, excluding the invalid copies. For Questionnaire 2, a total of 350 copies were sent out, with 306 retrieved copies.

Questionnaires 1 and 2 were designed using the Likert 5-point scoring method, including the 7 dimensions mentioned above related to entrepreneurial psychological capital. Respondents made choices from “fully compliant” to “completely non-compliant” according to their circumstances, which is noted as 1–5 points, respectively (Paschek et al., 2018). There is an internal differentiation between entrepreneurial venture capital and employee deviant innovation behaviors. To explore the relationship between them, considering the theme of this experiment, Questionnaire 1 for entrepreneurial venture capital and Questionnaire 2 for employee deviant innovation behaviors were designed. Questionnaire 1 included three dimensions, i.e., entrepreneurial psychological capital, entrepreneurial opportunity capacity, and entrepreneurial performance, with a total of 11 component dimensions. The specific composition of the Questionnaire 1 is shown in Table 1. Questionnaire 2 includes 5 dimensions of employee deviant innovation behaviors, and the specific composition is shown in Table 2 below. The scale of the company involved in this study includes small, medium, and medium-sized companies. The positions of the investigators include chairman, general manager, and middle and middle-level executives. Most of the people involved are aged 30–40 years old (born in the 1980s–1990s), who mainly have the college and undergraduate education background.

**TABLE 1:** Internal consistency coefficient of Questionnaire 1.

Category	Element	Cronbach’s alpha
Entrepreneurial psychological capital	Positive growth	0.800
	Actively respond to	0.771
	Enthusiasm for innovation	0.843
	Acute superior	0.805
	Self-efficacy	0.870
	Social wisdom	0.877
	Optimistic hope	0.817
	Total table	0.973
Entrepreneurial opportunity ability	Opportunity seeking ability	0.937
	Opportunity recognition ability	0.944
	Opportunity evaluation ability	0.912
	Total table	0.971
Entrepreneurial performance	Survival	0.861
	Grow up	0.896
	Total table	0.933

**TABLE 2:** Internal consistency coefficient of Questionnaire 2.

Element	Cronbach’s alpha
Deviant innovation	0.896
Psychological empowerment	0.894
Work value	0.901
Task interdependence	0.883
Emotional intelligence	0.869
Total table	0.972

The results of the internal consistency reliability study of Questionnaire 1 are shown in Table 1.

According to Table 1, the internal consistency reliability of the entrepreneurial psychological capital part of Questionnaire 1 is 0.973, the internal consistency reliability of the entrepreneurial opportunity capability part is 0.971, and the internal consistency reliability of the entrepreneurial performance part is 0.933. The above data indicate that Questionnaire 1 has good performance and reliability.

The results of the internal consistency reliability study of Questionnaire 2 are shown in Table 2.

According to Table 2, the internal consistency reliability of Questionnaire 2 is 0.972, indicating that Questionnaire 2 has good reliability.

## Results

### Survey Results of Psychological Capital of Entrepreneurship and Deviant Innovation Behavior

**FIGURE 3:**
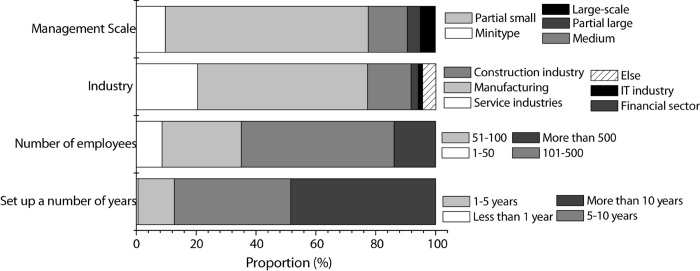
Company survey results of companies (year of company establishment; the number of company employees; industry in which the company operates; company scale of operation).

**FIGURE 4:**
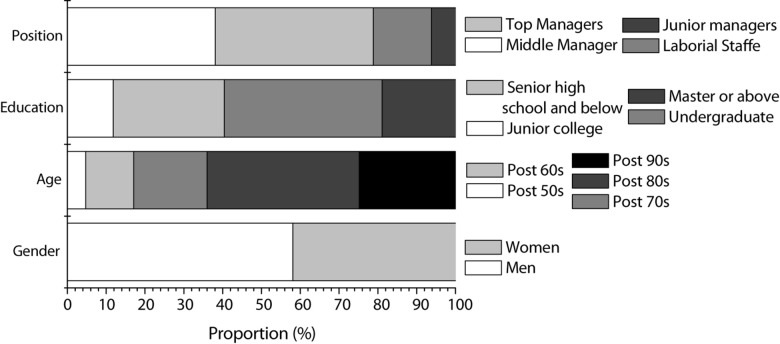
Staff survey results of Questionnaire 1 (gender; age; education; positions held).

The specific survey results of the companies are shown in Figure 3, and the specific survey results of entrepreneurial staff are shown in Figure 4.

According to Figure 3, for most of the companies surveyed in this study, the years of the establishment are more than 5 years, and the number of company employees is more than 50. The industry in which the company operates is mostly in the manufacturing industry, and most of the companies are small-scaled.

According to Figure 4, the proportion of male entrepreneurs is significantly higher than that of female entrepreneurs. At present, the age of entrepreneurs has become younger, and the educational level of entrepreneurs is mostly more than a college degree. Entrepreneurs also hold high positions within the company.

**FIGURE 5:**
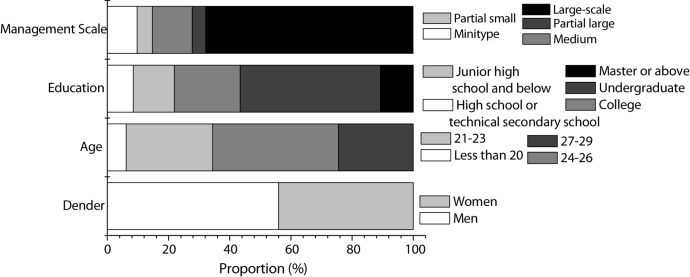
Survey results of Questionnaire 2 (gender; age; education level; company size).

The survey results of Questionnaire 2 are shown in Figure 5.

According to Figure 5, in terms of age, the employees involved in this study are generally younger, almost new-generation employees, and most of the employees participating in the study are born in the 1990s who have just joined the work. It seems that the research in this study covers people at all levels of education, which lays the foundation for the universality of the subsequent study.

### Research Results of Psychological Capital of Entrepreneurship

The research results of entrepreneurial opportunity ability and psychological capital of entrepreneurship of Questionnaire 1 are shown in Table 3.

**TABLE 3:** Results of analysis of variance in entrepreneurial opportunity capability and psychological capital of entrepreneurship.

Element	Age		Education		Position	
	*F*	*p*-value	*F*	*p*-value	*F*	*p*-value
Entrepreneurial psychological capital	36.265	0.000	40.084	0.000	29.401	0.000
Entrepreneurial opportunity ability	28.785	0.000	29.900	0.000	29.310	0.000

According to Table 3, there are significant differences in the psychological capital of entrepreneurship and entrepreneurial opportunity ability among people of different ages, with various educational backgrounds, and in distinct positions (*p* = 0.000).

Further analysis confirms that males have higher scores of psychological capital of entrepreneurship and the entrepreneurial opportunity ability than females, and the differences are statistically significant (*p* < 0.01). As the age decreases, the scores of the psychological capital of entrepreneurship and entrepreneurial opportunity ability show an increasing trend. With the increase in education, the scores of psychological capital of entrepreneurship and entrepreneurial opportunity ability have increased, the middle and senior managers have higher scores of psychological capital of entrepreneurship and entrepreneurial opportunity ability scores. However, the scores of chairman of the board in the psychological capital of entrepreneurship and entrepreneurial opportunity ability show a descending trend.

The correlation analysis has found that the correlation coefficient between entrepreneurial performance and entrepreneurial psychological capital is 0.29, *p* = 0.000, indicating that there is a significant correlation between entrepreneurial psychological capital and entrepreneurial performance. The correlation coefficient between entrepreneurial opportunity ability and entrepreneurial performance is 0.40, *p* = 0.000, indicating that there is a significant correlation between entrepreneurial opportunity ability and entrepreneurial performance. The correlation coefficient between entrepreneurial psychological capital and entrepreneurial opportunity ability is 0.81, *p* = 0.000, indicating that there is a significant correlation between entrepreneurial psychological capital and entrepreneurial opportunity ability. Therefore, the entrepreneurial opportunity ability plays an intermediary role between entrepreneurial psychological capital and entrepreneurial performance. Combined with Figure 1, Hypothesis 1 and Hypothesis 4 were verified, indicating that the hypotheses proposed are reasonable.

### Research Results of Deviant Innovation Behavior

By analyzing the results in Questionnaire 2, this study obtains the results, as shown in Table 4.

**TABLE 4:** Analysis results of Questionnaire 2.

Element	*F*	*p*-value
Work values and psychological empowerment	0.624	0.000
Work values and deviant innovation	0.367	0.000
Psychological empowerment and deviant innovation	0.367	0.000

According to Table 4, there are significant correlations between the work values and psychological empowerment of employees, the work values and deviance innovation of employees, and the psychological empowerment and deviance innovation of employees.

Further analysis shows that the scores of psychological empowerment and work values of employees during the period of low emotional intelligence show a downward trend, while the scores of the psychological empowerment of employees increase significantly when they show high emotional intelligence, and the differences are statistically significant (*p* = 0.000). When task interdependence is high, the psychological empowerment and deviance innovation scores of employees are lower, and the difference is statistically significant (*p* = 0.003). Therefore, employee psychological empowerment plays a role in regulating their work values and deviant innovation behaviors. Here, it is referred to as an intermediary role. In summary, considering the elaborations in Figure 2, the proposed Hypotheses 1–4 were verified.

## Discussion

Companies hope that employees may innovate to a certain degree and encourage employees to innovate. However, if the creative thinking of employee conflicts with the development goals of the company, they will be considered as performing deviant innovation behaviors. This study explores the psychological capital of entrepreneurship and deviant innovation behavior of employees separately (Zhu et al., 2019). After analyzing the research results, the authors found that there is a significant correlation among entrepreneurial psychological capital, entrepreneurial opportunity ability, and entrepreneurial performance; there is a significant correlation between entrepreneurial psychological capital and entrepreneurial opportunity ability; there are respective significant correlations between the work values and psychological empowerment of employees, between the work values and deviant innovation behaviors of employees, and between the psychological empowerment and deviant innovation behaviors of employees (Zheng and Liu, 2020). After research, it is found that the research results of this study are basically consistent with the pre-research hypotheses mentioned in the method section. It is not difficult to find that entrepreneurial psychological capital will have a great influence on employees’ deviant innovation behavior; meanwhile, there is a significant correlation between the two. In the meantime, the impact of employees’ working values on their deviant innovation behavior cannot be ignored. In general, working values can also be regarded as a manifestation of employees’ psychological empowerment, which further reveals the importance of entrepreneurial psychological capital in employees’ deviant innovation behaviors.

In addition, the research in this study has also found that the number of female entrepreneurs has a clear upward trend; however, the level of human capital and social capital of female entrepreneurs is lower than that of male entrepreneurs. As a result, the most important thing for female entrepreneurs is to improve their self-efficacy. Also, it is found that the average psychological capital of entrepreneurship is significantly improved for those with a history of starting a business than those without a history of starting a business. However, those with a history of starting a business twice do not have a clear advantage over those with a history of starting a business. Therefore, this proves that entrepreneurial experience cannot improve entrepreneurs’ ability of discovery and innovation. Through the research, it is found that age is a very important factor in deviant innovation behavior. Young employees are more eager to be recognized by company executives and colleagues, so their views on deviant innovation behavior are also simpler, and they are prone to deviant behavior. These results are consistent with the research results of Kluemper et al. (2019). The research on psychological capital and deviant innovation are enriched, which provides a reference for the practice of innovation management. Entrepreneurial self-efficacy is also a manifestation of mental state. In comparison, young people tend to show higher enthusiasm at work, with stronger impetus and innovation awareness. Thus, the possibility of deviant innovation behaviors among young people is higher. Based on gender considerations, the above results indicate that research on female entrepreneurs is necessary and valuable, which will also be the direction of further deepening research.

## Conclusion

In this study, the psychological capital of entrepreneurship and deviant innovation behavior of employees were studied in a separate manner. The research results show that there is a significant correlation between the psychological capital of entrepreneurship and entrepreneurial opportunity capability, a significant correlation between the psychological capital of entrepreneurship and entrepreneurial performance, and entrepreneurial opportunity capability and entrepreneurial performance. Also, there is a significant correlation between work values and the psychological empowerment of employees, work values and deviant innovation behavior of employees, and psychological empowerment and deviant innovation behavior of employees.

Although this study has obtained valuable results, the research process of this study still has the following limitations.

(1) The psychological capital structure used in this paper is the one commonly used in Europe and America; for China, however, this psychological capital structure may not be completely applicable. It is therefore necessary to change the structure of psychological capital of entrepreneurship, so as to fit better in China in the subsequent study.
(2) Due to the time limitation, This study did not focus on gender differences, which is of great value to female entrepreneurs. In the subsequent study, it is essential to conduct psychological research on the entrepreneurial capital of female entrepreneurs.
(3) The data analyzed in this study are filled out by individuals, and the accuracy cannot be guaranteed. In the future, relevant evaluation data from others will be added to consolidate the findings of this study.

## Data Availability Statement

The raw data supporting the conclusions of this article will be made available by the authors, without undue reservation.

## Ethics Statement

The studies involving human participants were reviewed and approved by the Hefei University of Technology Ethics Committee. The patients/participants provided their written informed consent to participate in this study.

## Author Contributions

QG: writing – original draft preparation. ZT: methodology and software. JX: formal analysis, resources, and data curation. CW: writing – review and editing. All authors contributed to the article and approved the submitted version.

## Conflict of Interest

The authors declare that the research was conducted in the absence of any commercial or financial relationships that could be construed as a potential conflict of interest.
